# Mesoporous-rich calcium and potassium-activated carbons prepared from degreased spent coffee grounds for efficient removal of MnO_4_^2−^ in aqueous media†

**DOI:** 10.1039/d2ra02214a

**Published:** 2022-07-04

**Authors:** Suranjana Bose, Rebecca D. Kirk, Harry Maslen, Martha A. Pardo Islas, Benedict Smith, Thomas I. J. Dugmore, Avtar S. Matharu

**Affiliations:** Green Chemistry Centre of Excellence, Department of Chemistry, University of York YO10 5DD UK suranjana.bose@york.ac.uk tom.dugmore@york.ac.uk avtar.matharu@york.ac.uk

## Abstract

Commercial ACs typically possess high surface areas and high microporosity. However, ACs with appreciable mesoporosity are growing in consideration and demand because they are beneficial for the adsorption of large species, such as heavy metal ions. Thus, in this study, degreased coffee grounds (DCG) were used as precursors for the production of ACs by means of chemical activation at 600 °C for the efficient removal of manganese in the form of MnO_4_^2−^. One of the most common activating agents, ZnCl_2_, is replaced by benign and sustainable CaCl_2_ and K_2_CO_3_. Three ratios 1 : 1, 1 : 0.5 and 1 : 0.1 of precursor-to-activating agent (g g^−1^) were investigated. Porosimetry indicates 1 : 1 CaCl_2_ DCGAC is highly mesoporous (mesopore volume 0.469 cm^3^ g^−1^). CaCl_2_ DCGAC and K_2_CO_3_ DCGAC shows high adsorption capacities of 0.494 g g^−1^ and 0.423 g g^−1^, respectively for the uptake of MnO_4_^2−^ in aqueous media. The adsorption process follows pseudo-second order kinetics inline with the Freundlich isotherm (*R*^2^ > 0.9). Thermodynamic data revealed negative values of Δ*G* (approx −0.1751 kJ mol^−1^) demonstrating that the adsorption process on 1 : 1 CaCl_2_DCGAC was spontaneous.

## Abstract 2

Spent coffee grounds are an interesting high volume renewable resource for valorisation. Activation with CaCl_2_ produces mesoporous carbons with high capacity for removal of MnO_4_^2−^.

## Graphical abstract

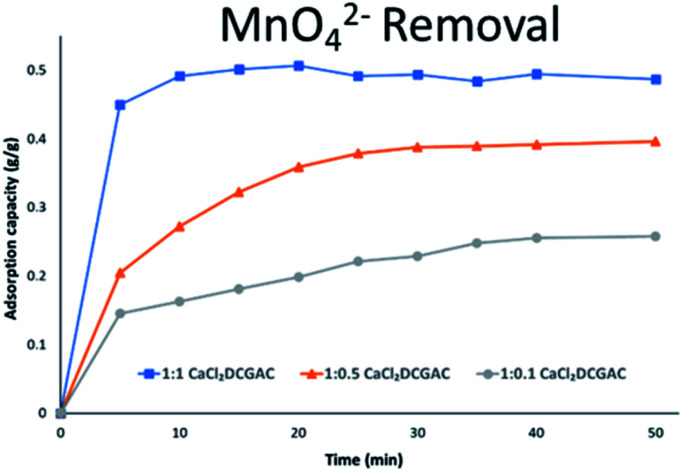

## 1 Introduction

Freshwater availability depletes as the demand for water grows in a higher proportion than the treatment rate. After being used, the quality of water is altered by the presence of pathogens and organic and inorganic chemicals that, in toxic concentrations, make water unsafe for human intake. One particular class of pollutant that is of particular interest are metal ions and salts. Many metal ions in water have harmful, or even toxic properties at very low (<1%) concentrations with most countries imposing strict limits on their concentration in both effluent and drinking water.^1,2^ The removal of many of these metals from waste water is a serious challenge for water treatment companies worldwide. Many methods for metal contaminant removal from water have been developed including chemical precipitation, ion-exchange, membrane filtration, coagulation–flocculation, flotation or electrochemical separation.^3–6^ Such methods are generally energy intensive and expensive, and others produce impure sludge as a waste which is difficult to handle, process or valorise.

At the same time, the shift towards clean energy technologies and other metal-dependent electronics have dramatically raised the demand of many elements – particularly transition metals and lanthanides, diminishing their availability. Based on current extraction rates and volumes of known reserves, elements such Zn, Se, Sn, Sb, Au, Ti and Pb are referred to as ‘critical elements’ conventional sources of critical elements are projected to be depleted within 5–20 years.^7^ Recycling metal-rich effluent or sludge from waste water treatment therefore presents a potential means to simultaneously address both of these issues. Coinciding with this growing metal demand is the problem of increasing amounts of unavoidable food supply-chain waste (UFSCW).^8^ This is an issue identified by the United Nations in the 17 Sustainable Development Goals (SDGs). In particular, SDG12, Target 12.3 states, “By 2030, to halve per capita global food waste at the retail and consumer levels and reduce food losses along production and supply chains, including post-harvest losses”.^9^ Therefore, material wherever possible should be sourced from waste streams, rather than virgin material, creating an economically favoured waste management route at the end of a material lifecycle. As an example of this, in the coffee industry approximately 6 billion kg of spent coffee grounds (SCGs) are produced annually as UFSCW.^10^ The common mode of waste management for UFSCW is either biomass burning which, in the case of SCGs, has negative connotations associated with NOx emissions, or aerobic digestion where water soluble ammonia generated from decomposition of SCGs can raise the pH, killing or inhibiting the methanogens that generate methane for renewable fuel. Hence a waste valorisation route for the management of UFSCW spent coffee grounds (SCGs) is highly desirable.

Previously, we reported the production of mesoporous activated carbon (AC) *via* zinc chloride activation derived from degreased spent coffee grounds (DCGs) in adsorbing gold and chromium from aqueous solutions.^10^ However, with zinc being a ‘Critical element’, coupled with the health/environmental risks associated with it, it is essential to look for alternative, more benign and sustainable activating agent such as metal chlorides (*e.g.* Ca, Na). Calcium- and sodium-based activating agents are particularly attractive due to their high availability compared to zinc. Also, the extraction of zinc requires high temperatures to extract the metal from impure ore, whilst calcium carbonate is available in the relatively pure form of limestone and can be extracted at low temperatures using sodium chloride in the Solvay process.^11,12^ The efficacy of the resultant material was tested for its ability to remove manganese (in the form of MnO_4_^2−^) from aqueous solutions.

Manganese was selected in part due it being a critical element under severe supply risk but also due to health and environmental risks it poses in effluent streams. Manganese has important functions in biological systems as it is required for many enzymatic processes. A guideline level of 0.05 mg L^−1^ or 0.1 mg L^−1^ in drinking water is typically adopted worldwide.^1^ However, an excess of manganese in the human body can lead to neurological disorders whilst it can cause stunted growth in plants.^13^ Manganese in water supply services may lead to clogging in the pipelines.^14^ Additionally, high amounts of manganese can be released to the environment from a range of industrial processes. Manganese is used in the production of alloys, batteries, glass, cleaning products and fireworks^13^ and is used as a catalyst in many redox reactions.^15^ Over 90% of all the manganese ores processed is used in steel manufacturing, mostly in the form of ferromanganese.^16^ Thus, mine tailings and steel processing wastewater are significant sources of manganese pollution. Due to its high solubility in both acid and neutral conditions, manganese is identified as one of the most difficult elements to remove from mine waters with concentrations ranging from 40–140 mg L^−1^ being reported.^17,18^ The lack of treatment of these wastes enable manganese to reach surrounding ecosystems, leading to environmental deterioration, where concentrations of manganese in local rivers have been reported to be as high as 200 mg L^−1^.^19^ The recovery of manganese from waste streams to reintroduce back into the manufacture and chemical industries therefore presents a valuable opportunity to simultaneously improve the sustainability of these industries whilst also reducing their environmental impact. Reported examples so far have included using KMnO_4_ doped ACs to oxidise ethylene,^20^ catalyse reforming of greenhouse gases^21^ and removal of formaldehyde from indoor air.^22^

The most stable oxidation state of manganese is +2, hence there has been a substantial amount of work on adsorption of Mn^2+^ compounds from aqueous media reported.^23–27^ However, despite being a prominent industrial oxidation agent, adsorption of MnO_4_^−^ is far less extensively covered with the aforementioned examples focussing primarily on the production and testing of the material for the end purpose, rather than the efficacy of the adsorption of MnO_4_^−^ to begin with. Herein we report high adsorption capacities of CaCl_2_ DCGAC and K_2_CO_3_ DCGAC for the uptake of MnO_4_^2−^ in aqueous media.

## 2 Experimental

### 2.1 Materials and methods

Commercial activated carbon, NORIT, and anhydrous ZnCl_2_ were purchased from Alfa Aeser. 99% KMnO_4_, CaCl_2_, K_2_CO_3_ and HCl (analytical grade) were purchased from Fischer Scientific. Spent coffee grounds were sourced from catering outlets at the University of York, oven dried (105 °C) and stored in air-tight polythene bags. All pH measurements were taken using a Jenway model 3505 pH meter and an epoxy-bodied pH electrode with the results shown in the ESI in Table S2.† The nitrogen adsorption–desorption isotherms were recorded at liquid nitrogen temperature (77 K) on a Micromeritics TriStar II Plus porosimeter using the Barrett–Joyner–Halenda (BJH) and Brunauer–Emmett–Teller (BET) method. All SCG and activated DCG samples were carbonised in a ThermoFischer Carbolite muffle furnace. A Genesys 150 UV-Vis spectrometer used for adsorption studies (*λ*_max_ 525 nm). A 0.00046 M (150 mL) concentration of solution was chosen for analysis and the change in concentration of MnO_4_^2−^ against time agitated with activated carbons (200 mg) was used to determine the adsorption at time *t* (*q*_*t*_) using eqn (1). This relates the amount of solid added to a known amount of solution, and its change in concentration. Where, *C*_0_ is the initial concentration in g mL^−1^, *C*_e_ is the concentration at equilibrium in g mL^−1^, *V* is the volume of solution in litres, and *W* is the quantity of adsorbent in grams. Aliquots (2 mL) were taken every 5 minutes over a 40 minutes interval and their UV-vis spectrum was recorded. The decrease of absorbance at *λ*_max_ 525 nm was used to determine uptake of MnO_4_^2−^.1
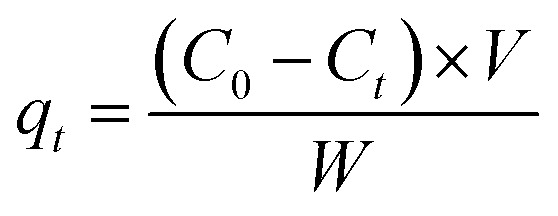


An Agilent 7700 series ICP-MS was used to conduct ICP-MS semi-quantitative analysis on the activated carbons before and after the adsorption studies. An Exeter Analytical Inc. CE-440 analyser was used to conduct Carbon, Hydrogen & Nitrogen (CHN) composition with the results shown in the ESI in Fig. S2.†

### 2.2 Production of degreased coffee grounds (DCG)

A mixture of dried spent coffee grounds (25 g) and ethyl acetate (100 mL) was heated to reflux for 2 h. The resulting slurry was cooled, filtered and the filtrate was evaporated *in vacuo* to dryness, whilst the residue was allowed to air dry until constant weight was achieved to afford the desired degreased coffee grounds (DCG), 21.67 g (86.7%), as a dark brown powder.

### 2.3 Chemical activation of DCG

Three ratios of DCG (10 g)-to-activating agent were investigated: 1 : 1, 1 : 0.5 and 1 : 0.1, corresponding to 10, 5, and 1 g of activating agent, respectively. The appropriate activating agent (ZnCl_2_ or CaCl_2_ or K_2_CO_3_) was weighed, dissolved in deionized water (DI) water (30 mL), added to DCG (10 g) and stirred at room temperature for 10 minutes. The slurry was dried at 105 °C for 24 h, ground into a fine powder and transferred to a quartz round-bottom flask to be carbonised. The flask was placed in the muffle furnace under nitrogen flow at 600 °C for 4 h. The resulting carbonised sample was ground into a powder, stirred with 0.5 M aqueous HCl (100 mL) for 10 minutes to remove ash and remaining activating agent, filtered under vacuum, washed with DI water (4 × 25 mL) until neutral pH and dried (105 °C for 24 h) until constant weight was achieved. The yield of DCGs were 22–25%.

## 3 Results and discussion

The important characteristic of every adsorbent is its specific surface area that greatly affects its adsorption capacity. The values of specific surface area, micropore volume (below 2 nm) and mesopore volume (2–50 nm) are good indicators of successful manufacture of the ACs before adsorption studies are conducted.

**Fig. 1:**
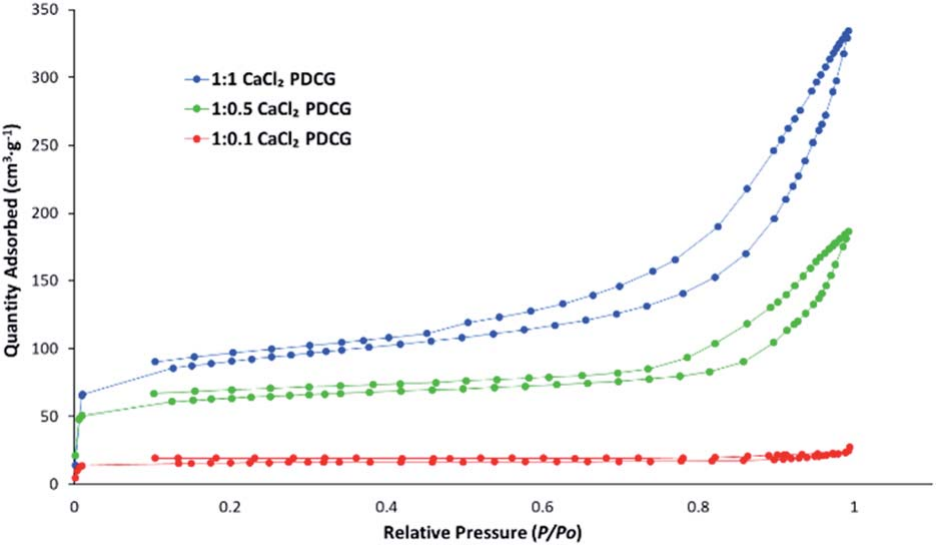
Nitrogen adsorption/desorption isotherm for the ACs at different ratios.

The nitrogen porosimetry data is summarised in Table 1 and Fig. 1 shows N_2_ adsorption–desorption isotherms for CaCl_2_ impregnated activated carbons. Except for the 1 : 0.1 K_2_CO_3_ DCGAC and non-treated (SCDCG and DCGAC), all chemically-treated activated carbons were successfully produced. The ZnCl_2_ (1 : 1 ZnCl_2_DCGAC) and K_2_CO_3_ (1 : 1 K_2_CO_3_DCGAC) activated carbons exhibited surface areas that are considered comparable with commercial NORIT (684 and 556 m^2^ g^−1^, respectively). The 1 : 1 CaCl_2_DCGAC showed a substantial increase in mesoporosity and mesopore volume compared to commercial activated carbon NORIT and in a higher proportion than 1 : 1 ZnCl_2_ DCGAC. Also surface area and total pore volume decreases as the amount of activating agent decreases. Hence 1 : 0.1CaCl_2_DCG has 91% less total pore volume than 1 : 1 CaCl_2_DCGAC (Fig. 1) and the chemically unactivated ACs (SCGAC and DCGAC) are non-porous.

**Table 1:** Porosimetry data

Activated carbon	Surface area (m^2^ g^−1^)	Micropore vol. (cm^3^ g^−1^)	Mesopore vol. (cm^3^ g^−1^)
NORIT	677	0.497	0.272
1 : 1 ZnCl_2_ DCGAC	684	0.234	0.526
1 : 1 CaCl_2_ DCGAC	305	0.142	0.469
1 : 0.5 CaCl_2_ DCGAC	205	0.103	0.223
1 : 0.1 CaCl_2_ DCGAC	50.8	0.030	0.022
1 : 1 K_2_CO_3_ DCGAC	477	0.253	0.105
1 : 0.5 K_2_CO_3_ DCGAC	556	0.291	0.134
1 : 0.1 K_2_CO_3_ DCGAC	2.74	0	0
SCGAC (unactivated)	0.63	0	0
DCGAC (unactivated)	0.38	0	0

The use of calcium chloride as an activating agent is less commonly seen in literature; therefore, the mechanism of its action is less decided. What is known though is that calcium chloride is hygroscopic and can form hydrated solids. That the DCGs were impregnated with a concentrated aqueous solution of calcium chloride before drying at 105 °C suggests a large presence of calcium chloride monohydrate and dihydrate (CaCl_2_(H_2_O)_*x*_, where *x* = 1, 2) in the sample before pyrolysis.^28^ These would undergo gasification at 600 °C, contributing to pore development in a similar manner to the gasification of potassium carbonate.

The mechanism of activation by potassium carbonate is well understood and documented in literature.^29,30^ Because potassium carbonate is a weak base, it cannot induce the same dehydrations as zinc chloride upon impregnation. At 600 °C, the temperature is sufficient to induce the decomposition of surface oxygen groups, releasing CO_2_ and CO.^31,32^ Throughout the pyrolysis step, resultant char (carbon) reduces the potassium carbonate to metallic K and CO, or the potassium carbonate undergoes degasification to K_2_O and CO_2_.^29,30^ The molten, metallic K is credited with expanding adjacent layers of the carbon network and developing porosity, and the evolution of the gases is effective at minimising tar formation within the structure.

**Fig. 2:**
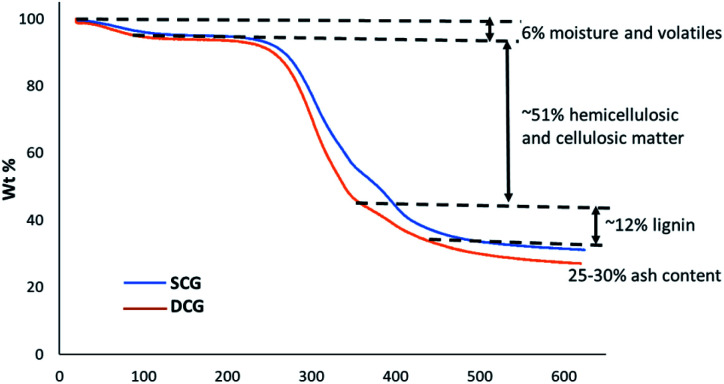
TG thermograms for SCG and DCG.

Fig. 2 shows the thermogravimetric analysis (TGA) for spent coffee grounds (SCG) and degreased coffee grounds (DCG) at the heating rate of 10 °C min^−1^ under nitrogen flow. A typical thermogram for the thermal decomposition of (ligno)cellulosic matter is observed. The thermogram reveals approx. 6% moisture and volatiles, 55–60% hemicellulosic and cellulosic matter and at 625 °C, approximately 25–30% of residual matter.

Table 2 shows the maximum amount of MnO_4_^2−^ adsorbed onto the DCGACs for each experiment, *q*_max_, using 0.00046 M KMnO_4_ solution alongside values obtained from literature for comparison.

**Table 2:** Maximum adsorption capacity of the DCGACs for MnO_4_^2−^ uptake

Activated carbons	*q*_max_ (MnO_4_^2−^) (g g^−1^)
NORIT	0.494
1 : 1 ZnCl_2_DCGAC	0.492
1 : 1 CaCl_2_DCGAC	0.494
1 : 0.5 CaCl_2_DCGAC	0.392
1 : 0.1 CaCl_2_DCGAC	0.256
1 : 1 K_2_CO_3_DCGAC	0.279
1 : 0.5 K_2_CO_3_DCGAC	0.423
1 : 0.1 K_2_CO_3_DCGAC	0.372
Unactivated DCGAC	0.173
Animal bone AC^33^	0.028
Coconut shell AC^20^	0.024
Commercial AC^34^	0.037
Corncob AC^33^	0.026

**Fig. 3:**
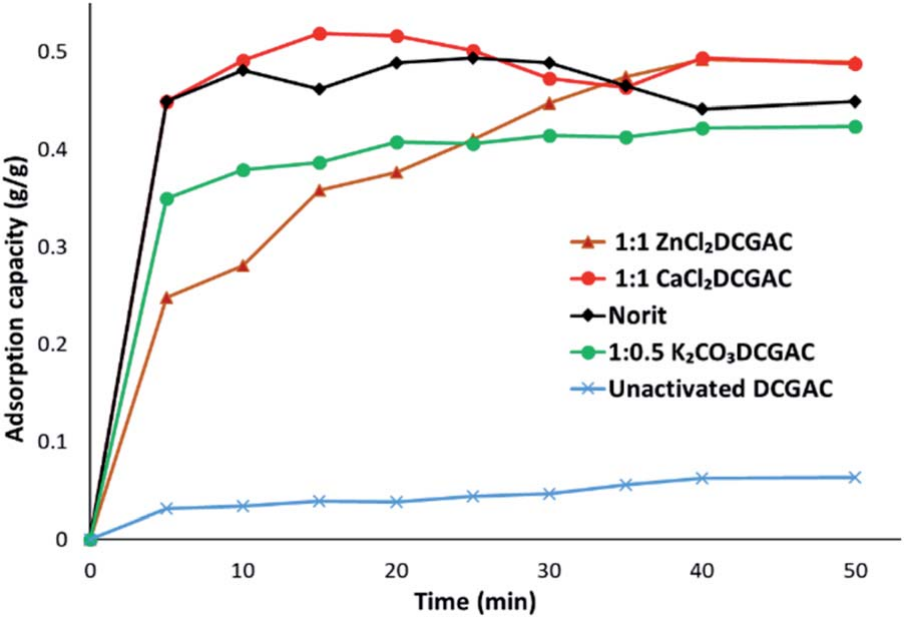
Adsorption capacities (*q*_*t*_) for 1 : 1 CaCl_2_DCGAC, Norit, 1 : 1 ZnCl_2_DCGAC, 1 : 0.5 K_2_CO_3_DCGAC and unactivated DCGAC when agitated with 0.00046 M KMnO_4_ solution at room temperature and atmospheric pressure.

Fig. 3 shows that 1 : 1 ZnCl_2_ and 1 : 1 CaCl_2_DCGACs have similar adsorptive properties to that of NORIT over a 40 minutes timeframe. NORIT was chosen as the commercial standard due to its high surface area (677 m^2^ g^−1^), high micropore volume (0.497 cm^3^ g^−1^) and appreciable mesopore volume (0.272 cm^3^ g^−1^).

**Fig. 4:**
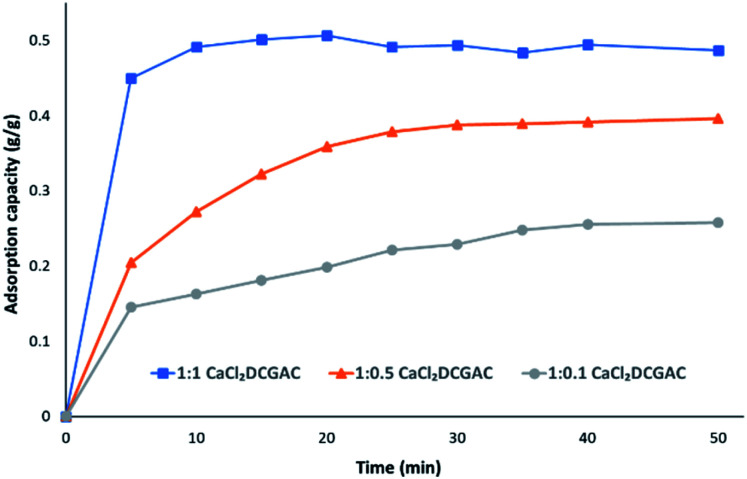
Adsorption capacity data for 1 : 1 CaCl_2_DCGAC, 1 : 0.5 CaCl_2_DCGAC and 1 : 0.1 CaCl_2_DCGAC agitated with 0.00046 M KMnO_4_ solution at room temperature and atmospheric pressure.

Fig. 4 shows the change in *q*_max_ of CaCl_2_DCGACs when varying the quantity of activating agent between ratios 1 : 1 (10 g DCG : 10 g activating agent), 1 : 0.5 (10 g DCG : 5 g activating agent) and 1 : 0.1 (10 g DCG : 1 g activating agent). This shows that *q*_max_ decreases as the amount of CaCl_2_ decreases, *i.e.*, 1 : 1 CaCl_2_DCGAC (*q*_max_, approx. 0.5 g g^−1^) > 1 : 0.5 CaCl_2_DCGAC (*q*_max_, approx. 0.4 g g^−1^) > 1 : 0.1 CaCl_2_DCGAC (*q*_max_, approx. 0.22 g g^−1^). This is to be expected given that the mesopore volume also decreases as the amount of CaCl_2_ (activating agent) decreases. Given that the Topological Polar Surface Area of the MnO_4_^−^ is computed to be 7.43 nm it would therefore be expected that it's adsorption onto surfaces would be affected more by the availability of mesopores (2–50 nm) than micropores. Hence 1 : 1 ZnCl_2_DCGAC and 1 : 1 CaCl_2_DCGAC show highest uptake of MnO_4_^−^ ions as they have considerably higher mesopore volume than other DCGACs (Table 1). This is further reflected by the fact that the values for MnO_4_^−^ on microporous activated carbons reported in Table 2 are of an order of magnitude lower. Notably, the *q*_max_ of the coconut shell AC was just 0.024 g g^−1^, despite using an AC that had a superior surface area of 1940 m^2^ g^−1^, but an average pore diameter of just 2.36 nm.^35^

The adsorption capacity was mapped onto pseudo-first and -second order kinetic theorems to investigate the rate of uptake of the contaminant using eqn (2) and (3) respectively.

2 *q_t_* = *q*_e_(1 − e^*k*_f_*t*^)

3
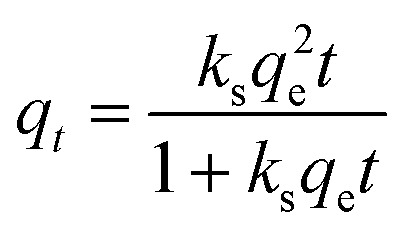
where *q*_*t*_ is the adsorption capacity at time *t*, *q*_e_ is the amount of adsorbates on the adsorbent surface when the reaction reaches equilibrium, *k*_f_ is the pseudo first order rate constant, *k*_s_ is the pseudo second order rate constant and *t* is the time in minutes.

**Fig. 5:**
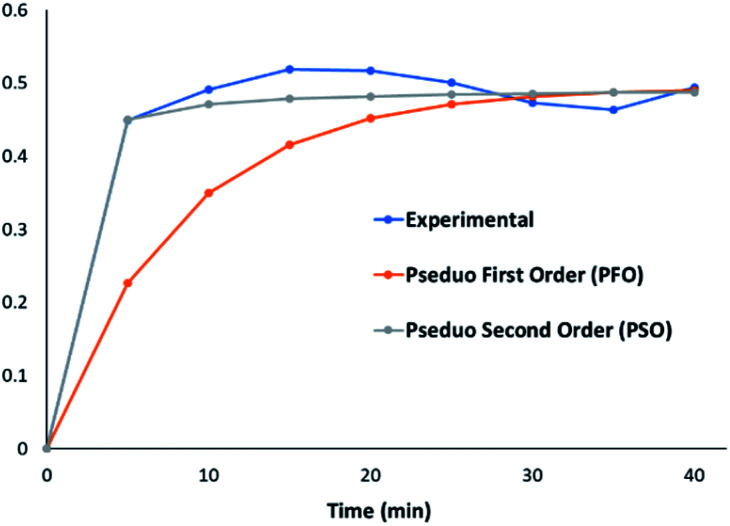
Kinetic modelling for the adsorption of MnO_4_^2−^ by 1 : 1 CaCl_2_DCGAC in 0.00046 M KMnO_4_ solution at room temperature.

Kinetic analysis (Fig. 5) was undertaken for the adsorption of MnO_4_^2−^ by 1 : 0.5 CaCl_2_DCGAC in 0.00046 M KMnO_4_ solution at room temperature. The data showed that adsorption follows pseudo second order kinetics at a near perfect precision to the theoretical prediction. This indicates that the adsorbent concentration is independent of the adsorption capacity of the adsorbent. The mechanism in the rate-limiting step is chemisorption, presumably entailing valence bonds between the metal ions and the materials surface.^36^

The strength of binding of the adsorbate and adsorbent was investigated using the Freundlich isotherm (eqn (4)), in particular its linearised form (eqn (5)).^37^4
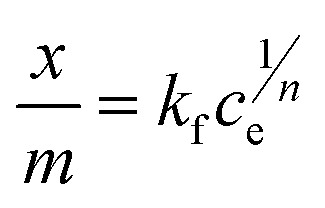
5
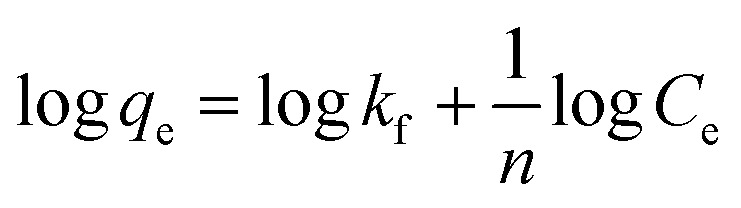
where *x*/*m* is the amount of adsorbed substance per gram of activated carbon (adsorption capacity) in g g^−1^, *C*_e_ is the equilibrium concentration in g L^−1^ and *k*_f_, *n* are specific constants.

**Fig. 6:**
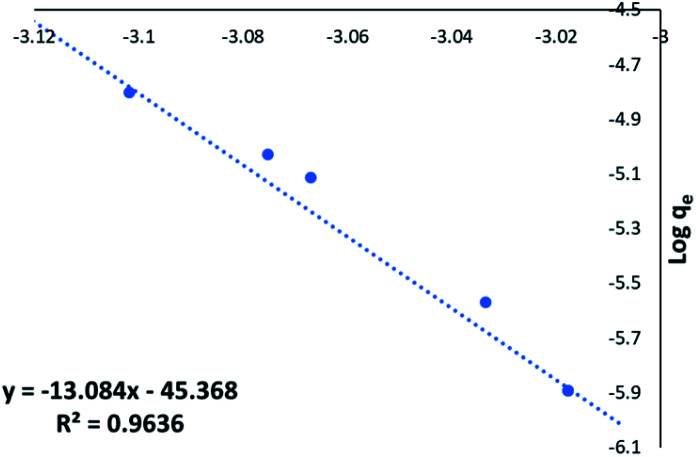
Freundlich plot for the MnO_4_^2−^ uptake by NORIT in 0.00046 M KMnO_4_.

The Freundlich plot for NORIT (Fig. 6) showed excellent linearity (*R*^2^ = 0.9636). The Langmuir isotherm was also investigated and is shown in ESI (Fig. S2†) but its regression was poorer (*R*^2^ = 0.7895). An excellent fit with the Freundlich model predicts strong adsorption to the adsorbent surface (NORIT) and the rate of binding is independent of the concentration of MnO_4_^2−^ ions in the adsorbent solution. Furthermore, it predicts a large capacity for adsorption by the material.

**Fig. 7:**
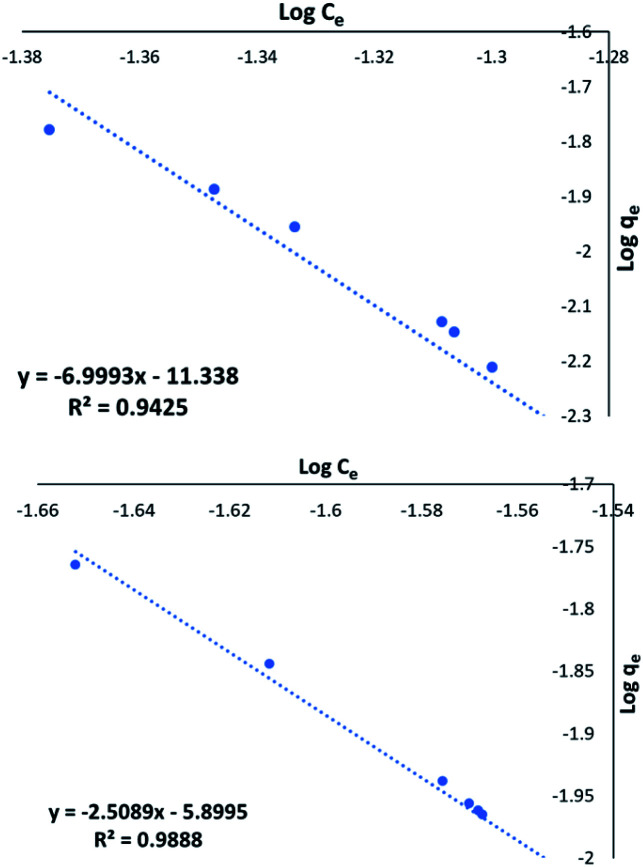
(a) Freundlich plot for the MnO_4_^2−^ uptake by 1 : 1 CaCl_2_DCGAC in 0.00046 M KMnO_4_, (b) Freundlich plot for the MnO_4_^2−^ uptake by 1 : 1 K_2_CO_3_ DCGAC in 0.00046 M KMnO_4_ solution.

Fig. 7a shows excellent linearity of the Freundlich plot for the 1 : 1 CaCl_2_DCGAC indicating strong adsorption and a match to the Freundlich theory, similar to NORIT. Therefore, the CaCl_2_ treated DCGACs are expected to have a strong binding mechanism with adsorbent, without any effect of the adsorbent concentration. Fig. 7b shows the Freundlich plot of the 1 : 1 K_2_CO_3_ DCGAC for MnO_4_^2−^ uptake. As seen previously, there is excellent linearity to this isotherm (*R*^2^ = 0.9888). This shows that these ACs follow the Freundlich isotherm with accurate proximity. Therefore, the binding of MnO_4_^2−^ ions onto the DCGAC is strong and the rate of binding is independent of the concentration of MnO_4_^2−^ ions in the adsorbent solution.

Thermodynamic studies were conducted to develop insight into the effect of temperature on the uptake of the adsorbent and to calculate thermodynamic parameters of the adsorption reactions. This effect was measured at 4 varying temperatures, 293 K, 308 K, 318 K and 328 K. The changes in concentration of the KMnO_4_ solution were examined and eqn (6) was used to determine the standard Gibbs free energy of each of the reactions occurring at the varying temperatures.^38^

6 Δ*G^θ^* = −*RT* ln *K*

where *R* is the molar gas constant in J mol^−1^ K^−1^, *T* is the temperature in Kelvin and *K* is the equilibrium constant in g^−1^ L^−1^ for the reaction. This data is then manipulated to denote the standard entropy and enthalpy for the given reaction using the vant Hoff equation shown by eqn (7).7
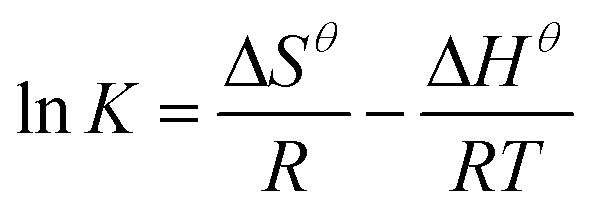
where *K* is the equilibrium constant in g^−1^ L^−1^, *R* is the molar gas constant in J mol^−1^ K^−1^ and *T* is the temperature in K. The entropy and enthalpy data are to be determined. As shown in Table 3, the obtained Δ*H* values were negative, which indicated the exothermic nature of adsorption. The change in entropy was positive, indicating the entropy of the system increased during the adsorption. The negative values of Δ*G* demonstrates that the adsorption process on 1 : 1 CaCl_2_DCGAC was a spontaneous process, and the increase of Δ*G* values with the increase of temperature indicated that the adsorption became less favourable at higher temperatures.

**Table 3:** Thermodynamic functions of MnO_4_^2−^ adsorption on 1 : 1 CaCl_2_DCGAC

*T* (K)	Δ*G* (kJ mol^−1^)	Δ*H* (kJ mol^−1^)	Δ*S* (J K^−1^ mol^−1^)
293	−0.1841	−0.2627	0.1418
308	−0.1751		
318	−0.1696		
328	−0.1645		

ICP-MS analysis of the DCGACs post adsorption studies gave an insight into the increase in the elemental concentration of the relevant elements from the solutions. Table 4 shows the increase in percentage of Mn after the adsorption studies clearly indicating a significant uptake of MnO_4_^2−^.

**Table 4:** ICP-MS analysis of DCGACs for Mn content before and after adsorption studies

Activated carbons	Mn content originally (%)	Mn content after MnO_4_^2−^ adsorption studies (%)
1 : 0.5 CaCl_2_DCGAC	Below detection limit	0.025
1 : 0.5 K_2_CO_3_ DCGAC	0.0019	0.040

## Conclusions

Remediation of manganese from effluent is both an environmental problem but also, an opportunity for resource recovery. Activated carbons and their production from CaCl_2_ and K_2_CO_3_ activated degreased spent coffee grounds can be successfully employed as an alternative to ZnCl_2_ to produce good quality ACs. Porosimetry indicates 1 : 1 CaCl_2_DCGAC is highly mesoporous (mesopore volume 0.469 cm^3^ g^−1^). CaCl_2_DCGAC and K_2_CO_3_DCGAC showed high adsorption capacities of 0.494 g g^−1^ and 0.423 g g^−1^ respectively for the uptake of MnO_4_^2−^ in aqueous medium.

As the consumption of filtered and brewed coffee continues to increase then the volume of this under-utilised resource will also increase. Although this study provides new insights in to the valorization of spent coffee grounds, its offering as a commercial activity will require a full techno-economic assessment, including security of supply and full life-cycle assessment.

## Author contributions

R. K., H. M., B. S. and M. P. I. investigation and methodology. S. B., investigation, methodology, writing the first draft and editing. T. I. J. D. & A. S. M. – funding acquisition, conception, supervision and final editing.

## Conflicts of interest

There are no conflicts to declare.

## Supplementary Material

RA-012-D2RA02214A-s001
