# Supplementary material for: Mesoporous-rich calcium and potassium-activated carbons prepared from degreased spent coffee grounds for efficient removal of MnO42− in aqueous media
Source: RSC Adv. 2022 Jul 4;12(30):19417–23. doi: 10.1039/d2ra02214a (PMC9252606; doi:10.1039/d2ra02214a)
Supplement: RA-012-D2RA02214A-s001 [file RA-012-D2RA02214A-s001.pdf]

Electronic Supplementary Material (ESI) for RSC Advances.
This journal is © The Royal Society of Chemistry 2022

Electronic Supplementary Information

Mesoporous-rich Calcium and Potassium-Activated Carbons Prepared from Degreased Spent Coffee Grounds for Efficient Removal of MnO_4^{2-} in Aqueous Media

Suranjana Bose,*[a] Rebecca D. Kirk,[a] Harry Maslen,[a] Martha A. Pardo Islas,[a] Benedict Smith,[a] Thomas I. J. Dugmore,*[a] and Avtar S. Matharu*[a]

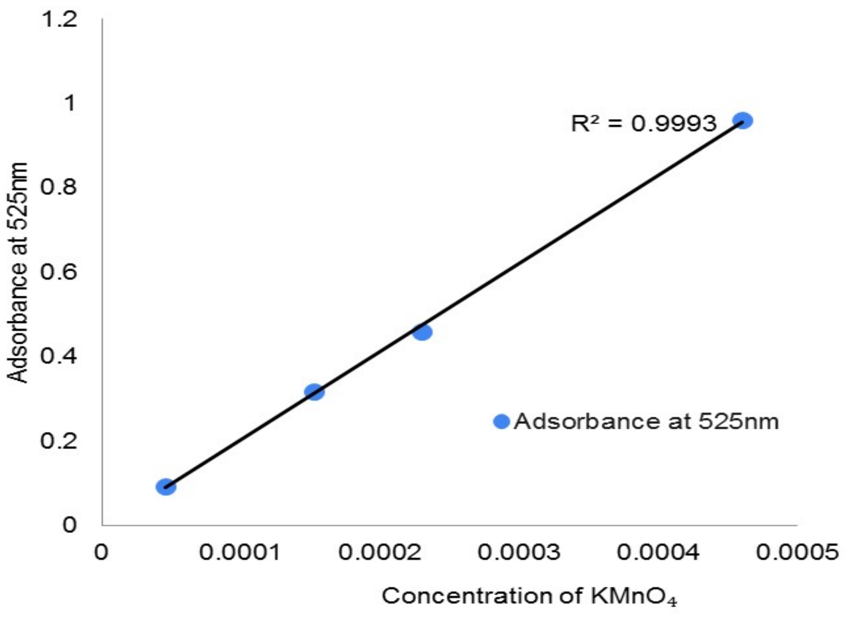

Figure S1. Calibration curve for the $KMnO_4$ solution

Table S1. Chemical composition of SCG in g per 100g dry material analysed[1]

Chemical Components	Composition (g/100g dry material)
Cellulose	12.4
Hemicellulose	39.1
Lignin	23.9
Fat	2.29
Ashes	1.30
Protein	17.4
Nitrogen	2.79
Total dietary fibre	60.5

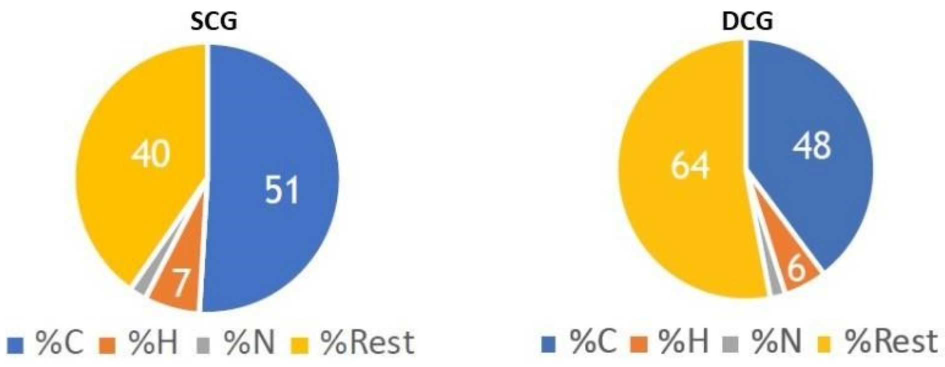

Figure S2. CHN analysis of SCG and DCG

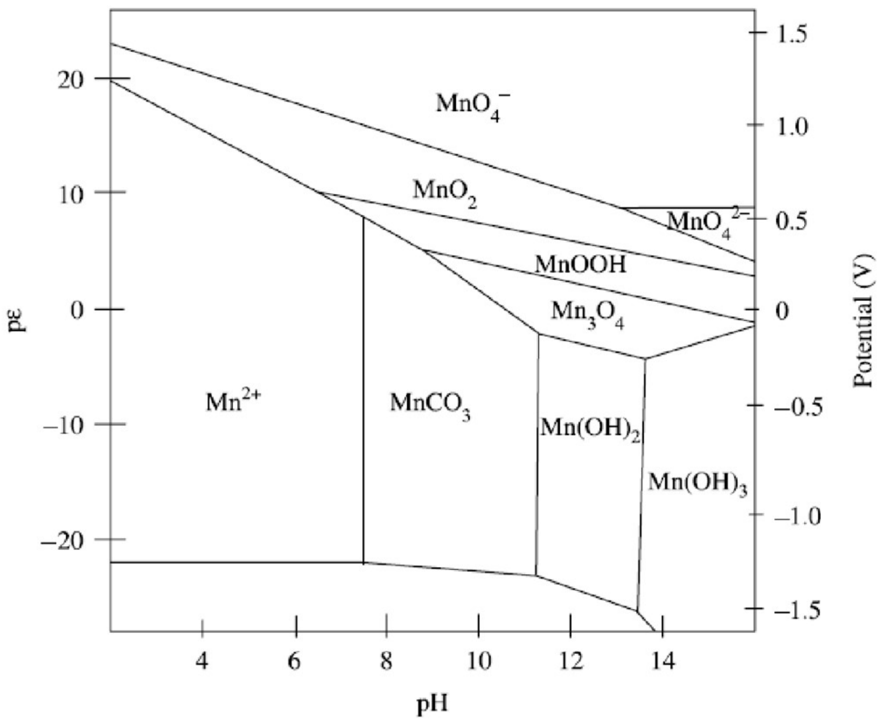

Figure S3. pε-pH diagram for aqueous Mn species[2]

Table S2 - THe pH of solutions with and without the AC and $KMnO_4$ added

	pH at 20°C
Water	7.02
1:1$CaCl_2$DCGAC + water	6.37
0.00046M $KMnO_4$ solution	5.75
0.00046M $KMnO_4$ solution + 1:1$CaCl_2$DCGAC	7.41

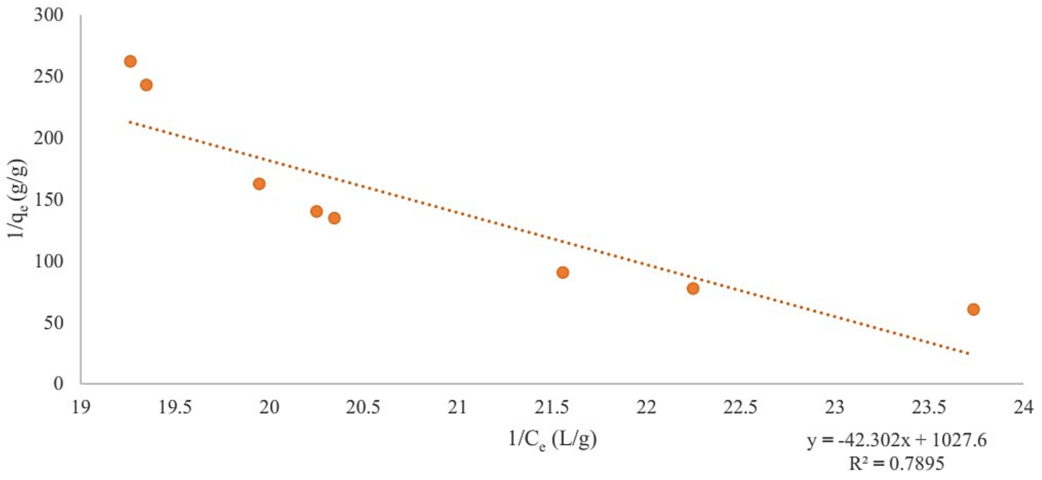

Figure S4. Langmuir modelling for 1:1 $CaCl_2$DGAC in 0.00046M $KMnO_4$ solution at room temperature.

References

1. L. F. Ballesteros, J. A. Teixeira, S. I. Mussatto, *Food Bioprocess Technol*, 2014, **7,** 3493.

2. W. Stumm and J. J. Morgan, *Aquatic Chemistry, Chemical Equilibria and Rates in Natural Waters*, 3rd Edition, John Wiley & Sons, Inc., New York, 1996, 462.
